# Deletion of integrin-linked kinase from neural crest cells in mice results in aortic aneurysms and embryonic lethality

**DOI:** 10.1242/dmm.011866

**Published:** 2013-06-05

**Authors:** Thomas D. Arnold, Keling Zang, Ainara Vallejo-Illarramendi

**Affiliations:** 1 Department of Pediatrics, University of California, San Francisco, CA 94143, USA; 2 Department of Physiology, University of California, San Francisco, CA 94158, USA

## Abstract

Neural crest cells (NCCs) participate in the remodeling of the cardiac outflow tract and pharyngeal arch arteries during cardiovascular development. Integrin-linked kinase (ILK) is a serine/threonine kinase and a major regulator of integrin signaling. It links integrins to the actin cytoskeleton and recruits other adaptor molecules into a large complex to regulate actin dynamics and integrin function. Using the Cre-*lox* system, we deleted *Ilk* from NCCs of mice to investigate its role in NCC morphogenesis. The resulting mutants developed a severe aneurysmal arterial trunk that resulted in embryonic lethality during late gestation. *Ilk* mutants showed normal cardiac NCC migration but reduced differentiation into smooth muscle within the aortic arch arteries and the outflow tract. Within the conotruncal cushions, *Ilk*-deficient NCCs exhibited disorganization of F-actin stress fibers and a significantly rounder morphology, with shorter cellular projections. Additionally, absence of ILK resulted in reduced *in vivo* phosphorylation of Smad3 in NCCs, which correlated with reduced αSMA levels. Our findings resemble those seen in *Pinch1* and β1 integrin conditional mutant mice, and therefore support that, in neural crest-derived cells, ILK and Pinch1 act as cytoplasmic effectors of β1 integrin in a pathway that protects against aneurysms. In addition, our conditional *Ilk* mutant mice might prove useful as a model to study aortic aneurysms caused by reduced Smad3 signaling, as occurs in the newly described aneurysms-osteoarthritis syndrome, for example.

## INTRODUCTION

In humans, cardiovascular and craniofacial malformations are among the most common birth defects. These pathologies result from a mixture of genetic and environmental interactions that often involve neural crest cells (NCCs), a pluripotent migratory population of cells that originate in the dorsal neural tube and participate in septation of the cardiac outflow tract (OFT) and remodeling of the pharyngeal arch arteries (Hiruma et al., 2002; Hutson and Kirby, 2003). Cardiac NCCs migrate from the dorsal neural tube through the pharyngeal arches in response to poorly understood signals (Creazzo et al., 1998). In the pharyngeal arch arteries, cardiac NCCs differentiate into smooth muscle cells, contributing to the smooth muscle layer covering the ascending aorta and derivatives of the aortic arch arteries. In the cardiac OFT, NCCs contribute to the aorticopulmonary septation complex that divides the OFT into the aorta and pulmonary trunk (Kirby et al., 1983).

Integrin-linked kinase (ILK) is a serine/threonine kinase and a major regulator of integrin signaling, interacting with the cytoplasmic domains of integrin β1 and β3 subunits. The main function of ILK is to link integrins to the actin cytoskeleton by recruiting other adaptor molecules into a large complex to regulate actin dynamics and integrin function (Legate et al., 2006; Hannigan et al., 2005; Wu, 2005). ILK together with PINCH and parvin form a heterotrimeric complex that recruits actin along with components of several signaling pathways to sites of focal adhesions. At the focal adhesions, ILK activity is crucial for modulating signaling pathways downstream of integrin β1, including PI3K-Akt-Rac1, which in turn regulate actin filament rearranging (Qian et al., 2005). In addition, ILK is able to induce phosphorylation of signaling factors such as Akt, GSK3 and PHI1 (Hannigan et al., 2005).

NCCs express multiple integrins that regulate NCC behavior *in vivo* and *in vitro* (Alfandari et al., 2003; Strachan and Condic, 2003; Thiery, 2003). Recent studies have highlighted the crucial role of β1 integrin, Pinch1 and Rac1 in NCC morphogenesis (Turlo et al., 2012; Liang et al., 2007; Thomas et al., 2010). It has recently been reported that loss of β1 integrin in NCCs results in early embryonic lethality due to pharyngeal arch artery aneurysms (Turlo et al., 2012). Interestingly, specific deletion of *Pinch1* and *Rac1* from NCCs using *Wnt1cre* mice results in a very similar cardiovascular phenotype, with abnormal remodeling of pharyngeal arch arteries, defective OFT septation and, most remarkably, the development of extremely dilated or aneurysmal aortic arch vessels (Liang et al., 2007; Thomas et al., 2010).

Human mutations in extracellular matrix (ECM) proteins, such as fibrillin 1 and collagen type III, can cause Marfan and Ehlers-Danlos syndromes. These disorders are characterized by aortic aneurysms, which are abnormal enlargements of the aorta caused by thinning of the vessel wall (Dietz et al., 1991; Dietz and Pyeritz, 1995; Pope et al., 1975). Also, genetic mutations in smooth muscle contractile proteins can result in hereditary vascular anomalies, such as patent ductus arteriosus, aortic aneurysms and aortic dissections (Guo et al., 2007: Pannu et al., 2007; Zhu et al., 2006). Also, mutations in genes encoding transforming growth factor beta (TGFβ) receptors (TGFBR1 or TGFBR2) have been shown to cause Loeys-Dietz syndrome, which is an autosomal dominant genetic syndrome with many features similar to Marfan syndrome, including ascending aortic aneurysms and dissections (Loeys et al., 2006). More recently, mutations in the gene encoding SMAD3, which is an essential effector of TGFβ signaling, cause the newly described aneurysms-osteoarthritis syndrome (AOS), which is characterized by aortic aneurysms with early-onset osteoarthritis (van de Laar et al., 2011; van de Laar et al., 2012). Therefore, development of aneurysms can result from abnormalities in vascular ECM proteins, smooth muscle cells, abnormal TGFβ signaling or a combination of these factors.

**TRANSLATIONAL IMPACT**

**Clinical issue**

Aortic aneurysms, abnormal enlargements of the aorta caused by thinning or weakness of the vessel wall, can cause severe pain, internal hemorrhage and even fatality if left untreated. These vascular anomalies, which are a feature of Marfan, Ehlers-Danlos and Loeys-Dietz syndromes as well as the newly described aneurysms-osteoarthritis syndrome, can be caused by mutations affecting the extracellular matrix, smooth muscle contractile proteins or Smad3 signaling factors. Another protein that is potentially implicated is integrin-linked kinase (ILK), a serine/threonine kinase with a role in integrin function and actin dynamics. Recent studies aiming to understand the underlying molecular mechanisms have focused on conditional mouse models in which mutations are specifically targeted to neural crest cells (NCCs), a migratory cell population that participates in the remodeling of aorta during cardiovascular development. Many of the NCC-specific mutants that have been developed so far display cardiovascular abnormalities; however, aortic aneurysms have only been described in a small number of mutants. The role of ILK in NCC morphogenesis and potentially in aortic aneurysm development has not yet been investigated.

**Results**

Using the Cre-*lox* system, the authors generated a conditional knockout mouse model with targeted *Ilk* deletion in NCCs during early development. The resulting mutant mice showed abnormal blood accumulation in the branchial arch region, and arterial abnormalities including a common arterial trunk (failure of the aorta and pulmonary artery to divide) and a highly aneurysmal common aortic sac. All of the mutants died during embryonic development, indicating that the conditional mutation is embryonically lethal. The authors then examined the effect of NCC-specific *Ilk* ablation on cell migration, proliferation and survival. These aspects of NCC function seemed normal; however, NCCs in mutant mice displayed a rounder morphology and abnormal actin cytoskeleton dynamics compared with wild-type NCCs. Interestingly, differentiation into smooth muscle in the outflow tract and phosphorylation of Smad3 (a process that is required for Smad3 signaling) were both reduced in the absence of *Ilk*.

**Implications and future directions**

These observations show that the ascending aortic wall is improperly configured in the absence of ILK in NCCs, providing genetic evidence that ILK signaling is essential for NCC morphogenesis and, in turn, aortic development. Moreover, the data suggest that disruption of ILK signaling in NCCs leads to aortic aneurysms through a signaling pathway that involves reduced Smad3 signaling. Thereby, *Ilk* conditional mutant mice might prove useful as a model to study aortic aneurysms caused by reduced Smad3 signaling, which is known to be a feature of aneurysms-osteoarthritis syndrome. Furthermore, the findings can be extended to other mouse models that have mutations in genes involved in integrin signaling, e.g. β1 integrin, *Pinch1* and *Rac1* conditional mutants. The phenotypic similarities among these mutant mice suggest that the β1 integrin signaling pathway involving ILK, Pinch1 and Rac1 is crucial for vascular function and might be altered in human disorders that are characterized by aortic aneurysms. Further studies are needed to elucidate the complete pathway involved, which could provide new targets for the development of therapeutic approaches.

To determine the role of ILK in NCC morphogenesis, we used the Cre-*lox* system to specifically delete *Ilk* from NCCs after embryonic day (E) 8.5. The resulting *Ilk* mutants developed a severe aneurysmal arterial trunk that resulted in embryonic lethality during late gestation. This phenotype closely resembles that seen in *Pinch1*, *Rac1* and β1 integrin conditional mutant mice; therefore, our results support that, in neural-crest-derived cells, ILK and Pinch1 act as cytoplasmic effectors of β1 integrin in a pathway that protects against aneurysms.

## RESULTS

### Specific ablation of *Ilk* from NCCs results in embryonic lethality

To investigate the function of ILK in cardiac NCCs *in vivo*, we crossed mice homozygous for a floxed allele of *Ilk* (*Ilk^flox/flox^*) with Wnt1cre; Ilk-floxed heterozygotes (Wnt1cre; *Ilk^flox/flox^*) mice to obtain specific *Ilk* inactivation in NCCs (Terpstra et al., 2003). The *Wnt1cre* transgenic mouse line has been previously used in numerous studies for targeted gene deletion and lineage tracing of NCCs. The *Wnt1* promoter is first activated at E8.5 in the neural crest and results in extensive Cre-mediated recombination in neural crest derivatives (Danielian et al., 1998). The neural crest origin of branchial arch mesenchyme was assessed in E10.5 embryos via the additional inclusion of the Cre-regulated *Rosa26lacZ* reporter allele (Soriano, 1999).

All *Wnt1cre; Ilk^flox/flox^* embryos died during embryonic development. *Ilk* conditional mutants were recovered at expected frequencies during mid-gestation (E10.5–E14.5), but only one was recovered during late gestation (E16.5–E20). No *Ilk* mutants were recovered after birth (Table 1).

**Table 1.**
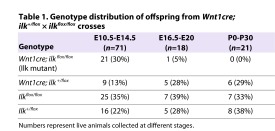
Genotype distribution of offspring from *Wnt1cre; ilk^+/flox^* × *ilk^flox/flox^* crosses

### Cardiovascular and craniofacial defects in *Wnt1cre; Ilk^flox/flox^* mutants

**Fig. 1.**
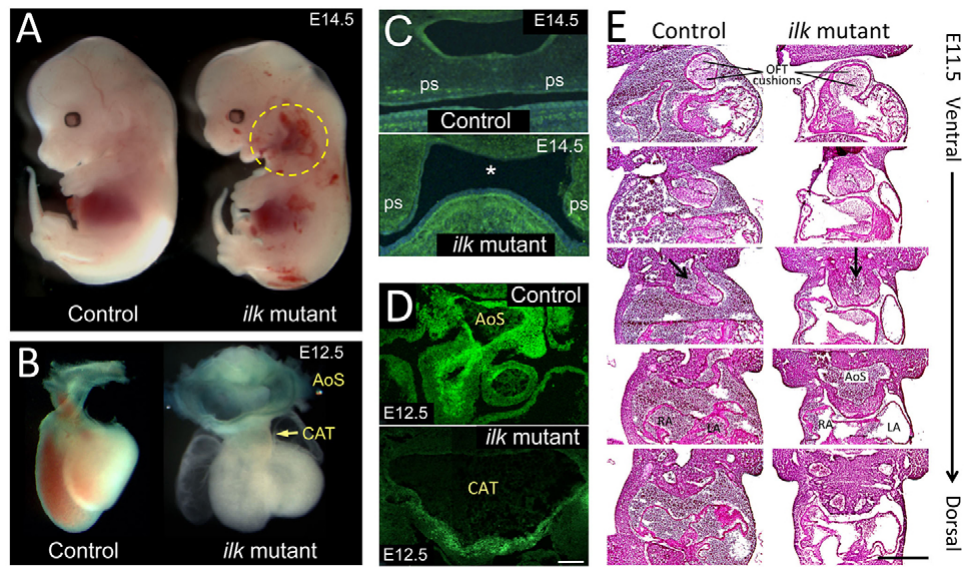
**Cranial and cardiovascular defects in *Wnt1cre; Ilk^flox/flox^* mice.** (A) Lateral view of a control and a mutant E14.5 embryo. *Ilk* mutants show aneurysms and hemorrhaging aortic arches (yellow circle). (B) At E12.5, cardiovascular development is severely affected in the conditional *Ilk* mice. Mutants display a common aortic trunk (CAT) and a highly dilated persistent aortic sac (AoS). (C,D) Frontal cryostat sections from E14.5 (C) and E12.5 (D) control and mutant mice stained for NCCs (βgal, green). *Wnt1cre; Ilk^flox/flox^* mice show misplaced palatal shelves (ps) that failed to rotate or elevate (asterisk) at E14.5. E12.5 sections show a highly dilated CAT that fails to septate in *Ilk* mutants. (E) Frontal serial sections from E11.5 control and mutant embryos stained for H&E that show impaired OFT rotation with abnormal positioning of the aorta (arrows) of *Ilk* mutants. Mutant embryos show normal formation of OFT cushions and, compared with controls, a much broader common vessel that extends from the aortic sac (AoS) to the 3rd aortic arch arteries. RA, right atria; LA, left atria. Scale bars: 100 μm (D), 250 μm (E).

At E9.5 and E10.5, conditional *Ilk* mutants did not exhibit any gross histological malformations. However, after E12.5, mutants could be recognized by abnormal accumulation of blood within the branchial arch region (Fig. 1A). By E12.5, all *Wnt1cre; Ilk^flox/flox^* embryos displayed a fully penetrant phenotype of a dilated common arterial trunk (CAT) with complete failure of OFT septation, and 42% of mutant embryos also showed ventricular septal defects. This CAT connected to a highly aneurysmal common aortic sac (AoS) that dilated very rapidly between E11.5 and E12.5 (Fig. 1B,D,E; Table 2). None of these malformations were observed in control littermates. E14.5 *Ilk* mutants also showed a cleft palate (*n*=2, Fig. 1C). Frontal sections of E14.5 embryos showed that, in contrast to control littermates, the palatal shelves of *Wnt1cre; Ilk^flox/flox^* embryos had neither rotated nor elevated. Frontal sections of E11.5 embryos also demonstrated abnormal OFT rotation with malposition of the aorta in *Ilk* mutants (arrows in Fig. 1E), and AoS dilatation was already noticeable by this stage.

**Table 2.**
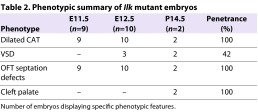
Phenotypic summary of *Ilk* mutant embryos

The observed cardiovascular and craniofacial malformations recapitulated common congenital defects that have been previously attributed to deficiencies in NCCs (Turlo et al., 2012; Liang et al., 2007; Thomas et al., 2010). The severe aneurysms within the aortic arches are very likely the cause of the embryonic lethality, because oxygen and nutrient supply must be severely compromised in the conditional *Ilk* mutants.

### Effect of *Ilk* deletion on NCC migration

**Fig. 2.**
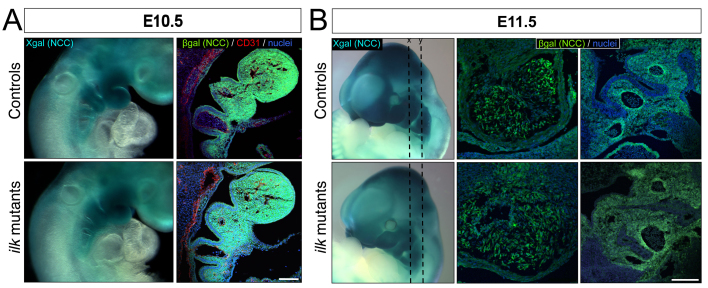
**Normal NCC migration to the aortic arches and cardiac OFT in conditional *Ilk* mutant embryos.** (A) Whole mounts of E10.5 X-gal-stained embryos (left panels) and sagittal sections (right panels) showing 1st and 2nd branchial arches of E10.5 embryos stained for NCCs (βgal, green), endothelium (CD31, red) and cell nuclei (DAPI, blue). (B) Whole mounts of E11.5 X-gal-stained embryos (left panels), where *x* and *y* are planes of sections for the middle panel (OFTs) and right panel (aortic arch arteries), respectively. Sections were stained for NCCs (βgal, green) and nuclei (DAPI, blue). Scale bars: 50 μm (A), 100 μm (B).

**Fig. 3.**
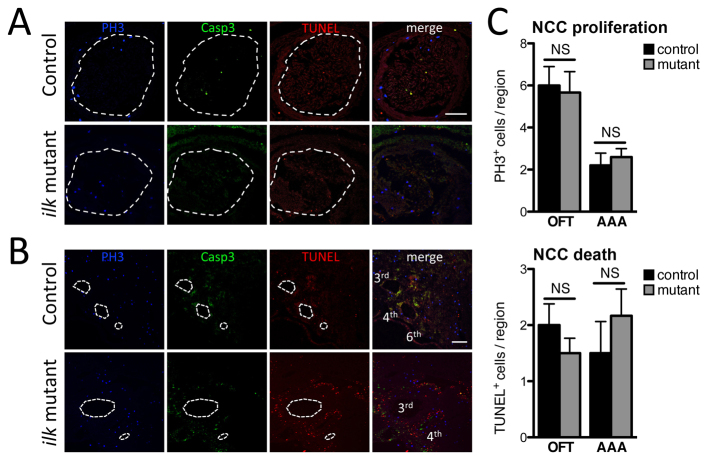
**Proliferation and death in E11.5 embryos.** (A,B) Frontal serial sections from control and conditional *Ilk* mutant embryos showing overall similar proliferation (phospho-histone H3, blue) and cell death (*In Situ* Cell Death Detection Kit, red) in neural-crest-derived cells within the cardiac OFT (A) and aortic arch arteries (B). Scale bars: 100 μm. (C) Quantification of these parameters did not show any significant changes in *Ilk*-deficient NCCs (*n*=4). However, in *Ilk* mutants, higher cell death was observed in specific areas adjacent to the 3rd aortic arch arteries (B). NS, non significant; AAA, aortic arch arteries; OFT, outflow tract.

We sought to determine whether the aortic arch patterning and OFT septation defects observed in the conditional *Ilk* mutants were due to defective NCC migration. To this end, we used the *R26R* reporter mouse (Soriano, 1999), in which Cre expression activates β-galactosidase. This mouse model enables us to follow NCCs as they migrate through the pharyngeal arch arteries, form the aorticopulmonary septum and differentiate into smooth muscle in the cardiac OFT. At E10.5 and E11.5, labeled NCCs were detected in the cranial, pharyngeal arch and trunk regions (Fig. 2). NCC migration appeared similar in conditional *Ilk* mutants and control littermates. We examined sagittal sections of E10.5 embryos. NCCs that migrate into the branchial arches were present in similar numbers and distributions in control and mutant embryos (Fig. 2A). Also, at E11.5, we found a similar distribution of NCCs within the conotruncal cushions (Fig. 2B). We also analyzed cell proliferation and cell death in E11.5 frontal sections (Fig. 3). Although we did not observe any significant differences between conditional *Ilk* mutant and control littermates in the NC-derived OFT or branchial arches, we did observe specific areas around the 3rd aortic arch artery that showed higher cell death as compared with control samples. Overall, our data indicate that initial specification and migration of *Ilk*-deficient NCCs is not altered in early cardiovascular development. It also indicates that there is no major alteration in *Ilk*-deficient NCC proliferation or survival in the neural-crest-derived OFT or branchial arches at this stage. Higher apoptotic levels detected in areas bordering the 3rd aortic arch artery are probably related to the ongoing enlargement of the aortic sac (see Fig. 1E; Fig. 3).

### Effect of *Ilk* deletion on NCC differentiation and TGFβ signaling

**Fig. 4.**
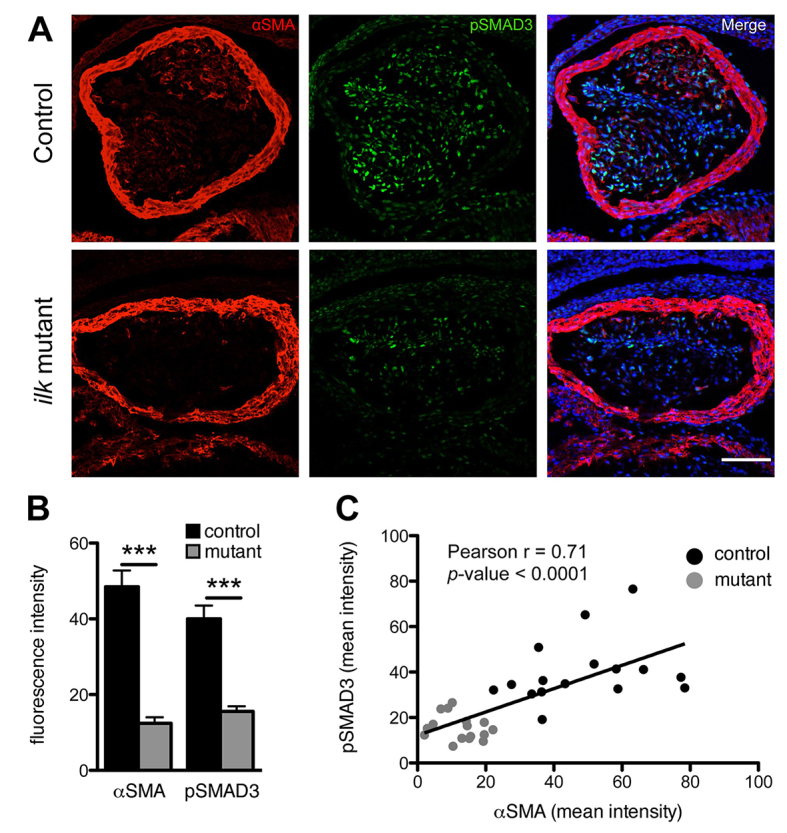
**Reduced NCC differentiation and Smad3 signaling in conditional *Ilk* mutants.** (A) Frontal sections of E11.5 cardiac OFTs triple stained for smooth muscle (αSMA, red), phospho-SMAD3 (pSMAD3, green) and nuclei (SYTOX, blue) as indicated. Scale bar: 100 μm. (B) Quantification of fluorescence intensity showed that conditional *Ilk* mutants display significantly reduced expression of both smooth muscle actin and phospho-SMAD3 in the neural-crest-derived cushions (*n*=4, ***P<0.001). (C) Levels of αSMA show a positive correlation with phosphorylated SMAD3 levels in the OFT cushions (Pearson *r*=0.71; *n*=32 sections; *P*-value <0.0001).

**Fig. 5.**
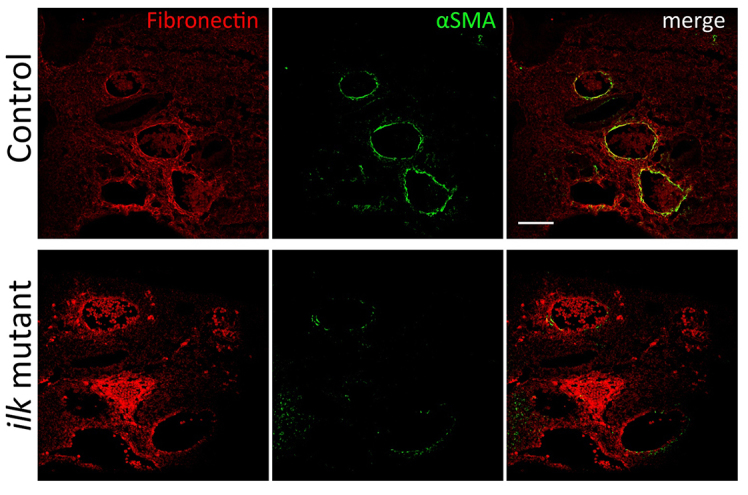
**Representative confocal images of E11.5 frontal sections showing reduced smooth muscle differentiation of NCCs but normal fibronectin expression in aortic arch arteries.** *n*=4 control and 4 mutant embryos analyzed. Scale bar: 100 μm.

To determine whether the cardiovascular defects observed in the *Wnt1cre; Ilk^flox/flox^* mutants were caused by defective differentiation of NCCs into smooth muscle cells, we analyzed the expression of smooth muscle actin (αSMA), a broadly used marker of smooth muscle differentiation. Previous studies have shown that failure of cardiac NCCs to differentiate into smooth muscle result in cardiovascular and craniofacial defects (Liang et al., 2007; Kochilas et al., 2002; Wurdak et al., 2005). Indeed, we found that αSMA is significantly reduced in *Ilk*-deficient NCCs within the conotruncal cushions at E11.5, as compared with controls (Fig. 4A,B). In the neural-crest-derived areas within the aortic arches, we also observed reduced αSMA expression but unchanged fibronectin levels, as compared with controls (Fig. 5). Our findings were further supported by a concomitant reduction in the levels of Smad3 phosphorylation, an essential mediator of TGFβ signaling, in *Ilk*-deficient conotruncal NCCs (Fig. 4A–C). These results are in line with the ones obtained with the *Pinch1* mutant (Liang et al., 2007) and indicate that ILK is required for correct TGFβ signaling and NCC differentiation into smooth muscle cells. Because deficient Smad3 signaling in humans results in aortic aneurysms, we propose that NCC differentiation is probably contributing to the aneurysmal CAT phenotype, which is also a common feature in *Ilk* and *Pinch1* mutants.

### Abnormal NCC morphogenesis in *Ilk* mutant OFTs

**Fig. 6.**
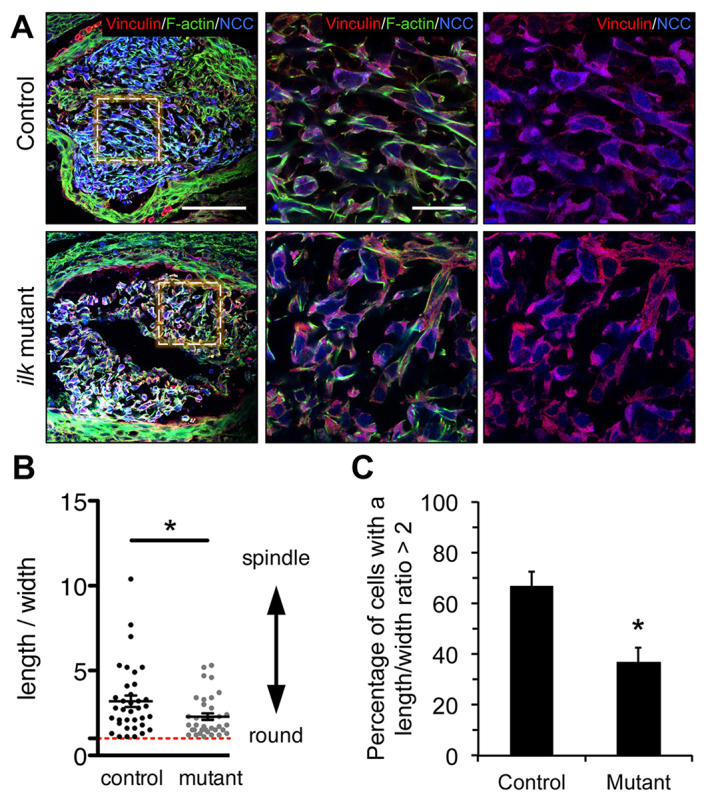
**Abnormal actin cytoskeleton dynamics and NCC morphology in *Ilk* mutant OFTs.** (A) Frontal sections of E11.5 cardiac OFTs triple stained for vinculin (focal adhesions, red), phalloidin (filamentous actin, green) and βgal (NCCs, blue). Compared with control, *Ilk*-deficient NCCs (blue) show normal focal adhesion formation, but a rounder cell shape and disorganized actin cytoskeleton. Left panel depicts lower-magnification images of triple-stained sections. Scale bar: 100 μm. Middle and right panels show higher magnification from the boxed regions. Scale bar: 25 μm. (B) Quantification of NCC elongation in OFT cushions showed significant reduction of the length:width ratio of *Ilk*-deficient cells, which appear more often rounder, with shorter cellular projections, than the corresponding control cells (*P<0.05). (C) Bar chart shows significantly lower numbers of *Ilk*-deficient NCCs with a length:width ratio >2 (*n*=4; *P<0.05).

Next, we examined focal adhesions and actin cytoskeleton dynamics in E11.5 OFT cushions by analyzing the expression pattern of vinculin and filamentous actin (Fig. 6). Our results show that the formation of focal adhesions is not grossly affected in *Ilk*-deficient NCCs, as demonstrated by normal vinculin distribution (Fig. 6A, red). However, we observed a reduction of actin stress fibers (Fig. 6A, green) and deficient NCC formation of condensed mesenchyme in mutant OFT cushions. These results are in line with observations in *Fak*-deficient NCCs (Vallejo-Illarramendi et al., 2009) and, thus, they are probably related to the abnormal OFT rotation and aorticopulmonary septation phenotypes, which are common features in both *Ilk* and *Fak* mutant mice. We also performed a detailed evaluation of NCC shape in the OFT cushions of four control and four mutant E11.5 embryos. This analysis showed that, compared with controls, *Ilk*-deficient NCCs had a significantly rounder morphology due to shorter or absent processes (Fig. 6B). Likewise, OFT cushions in *Ilk* mutants showed that significantly fewer cells had a length:width ratio higher than 2, as compared with control embryos (Fig. 6C). Interestingly, specific deletion of *Rac1* in NCCs results in an aneurysmal CAT by E12.5, and *Rac1*-deficient NCCs also display higher numbers of rounded NCCs in the OFT cushions at E11 (Thomas et al., 2010), which suggests that Rac1 might be an effector or modulator of ILK signaling pathways in NCC morphogenesis.

## DISCUSSION

In this study we demonstrate an important role of ILK in NCC morphogenesis. Mice lacking ILK in the neural crest developed craniofacial and cardiovascular malformations that led to embryonic lethality. Mutants displayed cleft palate together with several cardiovascular defects, including a highly aneurysmal aortic sac and CAT. Cardiac NCC migration seemed normal, but differentiation into smooth muscle in the OFT was reduced. In addition, absence of ILK resulted in reduced *in vivo* phosphorylation of Smad3, which is involved in a signaling pathway that is crucial for NCC development. Furthermore, NCC in the OFT region of *Ilk* mutants displayed abnormal actin cytoskeleton dynamics and a more rounded cell morphology, as compared with control embryos.

Murine mutants have shown that defects in multiple signaling pathways affecting cardiac NCC survival, migration or differentiation prevent normal development of the aortic arch arteries and cardiac OFT (Stoller and Epstein, 2005). Although there are many mouse models that present OFT septation failure and a CAT, the development of an extremely dilated aortic sac distinguishes *Ilk* mutants from many other NCC-specific mutants. Interestingly, embryos lacking Pinch1 and Rac1 in NCCs display a similar dysmorphic artery phenotype (Liang et al., 2007; Thomas et al., 2010). Also, aortic arch aneurysms have been reported in *Wnt1cre; β1 integrin* mice, which are embryonic lethal by E12.5 (Turlo et al., 2012). Both ILK and Pinch1 are known downstream mediators of β1 integrin, and numerous studies have implicated Rac1 in β1 integrin signaling (Berrier et al., 2002; Yu et al., 2005; Jeanes et al., 2012). This, together with the strikingly similar phenotypes of the NCC-specific mutants, suggests that these molecules are involved in a common signaling pathway during NCC morphogenesis that is crucial for vascular function and provides protection against aortic aneurysms.

Our results indicate that NCCs lacking ILK migrate normally, which differs from prior studies demonstrating β1 integrin involvement in NCC migration *in vivo* and *in vitro* (Alfandari et al., 2003; Kil et al., 1996; Kil et al., 1998). However, our results are consistent with a recent study showing that β1 integrin gene deletion from NCCs does not result in abnormal migration (Turlo et al., 2012). Also, as we showed in a previous work, NCC-specific deletion of *Fak*, which encodes a cytoplasmic tyrosine kinase activated by β1 integrin, does not grossly affect cardiac NCC migration (Vallejo-Illarramendi et al., 2009). Furthermore, neural crest ablation of Pinch1, a scaffold protein associated with integrins and integrin-linked kinase, does not preclude initial cardiac NCC migration to the pharyngeal arch region (Liang et al., 2007). Thus, it seems that β1 integrin signaling is not required for initial NCC migration to the branchial arches during development.

In conditional *Ilk* mutants, we found that NCC differentiation into smooth muscle is significantly decreased in the conotruncal cushions, and it is also relatively reduced in localized regions within the aortic arch arteries. Our results are consistent with the ones in the *Pinch1* conditional mutant mice, which display reduced NCC differentiation into smooth muscle mostly in the conotruncal cushions. Vascular aneurysms are seldom found in mice with impaired cardiac NCC differentiation (Kochilas et al., 2002; Wurdak et al., 2005; Vallejo-Illarramendi et al., 2009; High et al., 2007). Therefore, it is unlikely that a deficient NCC differentiation is the sole cause for aneurysms in the aortic arch region. Furthermore, deletion of β1 integrin and *Rac1* genes in the neural crest generates phenotypes highly reminiscent of those obtained by *Ilk* ablation, without affecting overall NCC differentiation to smooth muscle cells (Turlo et al., 2012; Thomas et al., 2010).

Recent studies show that mutations in Smad3 cause AOS, a new syndromic form of thoracic aortic aneurysms and dissections characterized by arterial aneurysms, early-onset osteoarthritis and mild craniofacial features (van de Laar et al., 2011; van de Laar et al., 2012). Interestingly, here we show that conditional *Ilk* mutants have reduced phosphorylation levels of Smad3 in the OFT cushions, which correlated with reduced αSMA expression levels. Similarly, reduced Smad2/3 phosphorylation levels are found in the OFT cushions of *Pinch1* mutants (Liang et al., 2007). Taken together, this suggests that disruption of ILK-Pinch1 signaling in NCCs results in reduced Smad3 phosphorylation that ultimately causes the aortic aneurysms phenotype. In the future, it will be interesting to determine whether Smad3 signaling is also reduced in β1 integrin and *Rac1* mutant mice. Although further studies are still needed to elucidate the complete pathway implicated in the unique aneurysmal phenotype of these mutants, evidence points to a β1-integrin–ILK–Pinch1 signaling pathway alteration that involves reduced phosphorylation of Smad3. Furthermore, the abnormal NCC morphology and actin cytoskeletal features shared by conditional *Ilk* and *Rac1* mutant mice further support that *Rac1* might be acting as a downstream effector or modulator of ILK signaling pathways in NCC morphogenesis.

In conclusion, our results demonstrate that the presence of ILK in NCCs is required for appropriate cardiac OFT morphogenesis and aortic arch remodeling. Based upon published results and our own, we propose that β1 integrin, ILK, Pinch1, Rac1 and Smad3 participate in a common pathway involved in an NCC morphogenetic program during cardiovascular development that, when perturbed, results in aneurysms in the ascending aorta. In the future, it will be interesting to understand how these molecules interact and contribute to the protection against aneurysms. Additionally, our conditional *Ilk* mutant mice might prove useful as a model to study aortic aneurysms caused by reduced Smad3 signaling, such as in the new AOS.

## MATERIALS AND METHODS

### Mouse lines

The *Ilk* floxed (*Ilk^flox/flox^*), R26R and *Wnt1cre* mice have been described previously (Terpstra et al., 2003; Soriano, 1999; Danielian et al., 1998). Mice were maintained on mixed genetic backgrounds. *Wnt1cre; Ilk^flox/flox^* mutants were compared with control littermates (*Ilk^+/flox^*, *Ilk^flox/flox^*, *Wnt1cre; Ilk^+/flox^*). Genotyping for *Cre* and *Ilk* alleles was determined by PCR as previously described (Danielian et al., 1998; Gheyara et al., 2007). Embryos were staged with the day of plug being E0.5. All animals were handled in accordance with protocols approved by the UCSF Animal Care and Use Committee.

### Reagents

Antibodies were obtained from the following sources: ILK polyclonal antibody (pAb) (A17; sc-557) (Santa Cruz Biotechnology); αSMA monoclonal antibody (mAb) (clone 1A4, A5228) (Sigma-Aldrich); phospho-histone H3 pAb (Upstate); phospho-SMAD3 mAb (Epitomics); CD31 mAb (clone MEC13.3, 550274) (BD Pharmingen), βgal pAb (Molecular Probes, A11132); vinculin mAb (clone hVIN-1, V9131) (Sigma-Aldrich); active caspase-3 pAb (Abcam, ab2302); fibronectin pAb (Abcam, ab2413). Filamentous actin was visualized using Alexa-Fluor-488-conjugated phalloidin (Invitrogen). Cell death was determined with the *In Situ* Cell Death Detection Kit, TMR red (Roche Products). Cell nuclei were labeled with DAPI (Sigma-Aldrich) or SYTOX Blue (Invitrogen).

### Histological analysis

Embryos were fixed in 4% paraformaldehyde for 3–4 hours at 4°C and stained for β-galactosidase activity resulting from Cre-mediated recombination of the *Rosa26lacZ* reporter allele using a standard protocol. Briefly, embryos were rinsed three times in rinse buffer (2 mM MgCl_2_, 0.01% sodium deoxycholate, 0.02% NP40; 5 mM EGTA in PBS) and staining was performed overnight at room temperature in staining buffer [1 mg/ml X-gal; 5 mM KFe_4_(CN)_6_; 5 mM KFe_3_(CN)_6_ in rinse buffer].

### Immunohistochemistry

Embryos were fixed in 4% paraformaldehyde overnight and transferred to 30% sucrose in PBS overnight. Afterwards, embryos were embedded in OCT and sectioned at 12 μm. Cryosections were preincubated with 2% normal goat serum, 5% BSA, 0.5% Triton X-100 in PBS for 1 hour. Slides were incubated overnight at 4°C with primary antibodies and then washed and incubated with Alexa-Fluor-488, -555 or -647-conjugated secondary antibodies (1:200; Molecular Probes). Confocal imaging was performed on a Zeiss LSM5 Pascal microscope.

### Quantifications and statistical analyses

All quantifications were done using high-resolution confocal images representing a thin optical section of the sample. Images were analyzed using ImageJ version 1.34s software. Each group contained four mutants and four littermate controls. Phospho-histone 3 and TUNEL-labeled NCCs were counted in sections with identifiable OFT cushions and in walls of aortic arch arteries. Quantification of phospho-SMAD3 (pSMAD3) and αSMA immunofluorescent staining was determined using stained cryosections from four mutants and four controls at E11.5. Within the cardiac OFT cushions, four randomly chosen confocal images were taken from each sample using the same confocal settings. ImageJ software was used to quantify the intensity of staining in each image and these values were averaged for each group. *P*-values were defined using Student’s *t*-test for paired comparisons. Correlation between pSMAD3 and αSMA immunofluorescent staining levels in the OFT cushions was assessed by Pearson’s correlation test (*n*=32 images analyzed from four control and four mutant E11.5 embryos). For cell shape studies, 36 cells were selected randomly from four separate high-resolution images per OFT and analyzed using ImageJ software. Each analysis was performed with four independent samples. *P*-values of less than 0.05 were considered statically significant. Data are expressed as mean ± s.e.m.
